# Use of an augmented reality headset to optimize ergonomics in digestive endoscopy settings

**DOI:** 10.1055/a-2695-4138

**Published:** 2025-09-11

**Authors:** Jean Grimaldi, Elena De Cristofaro, Julien Isman, Florent Morin, Louis-Jean Masgnaux, Jérôme Rivory, Mathieu Pioche

**Affiliations:** 1 Gastroenterology and Endoscopy Unit, Edouard Herriot Hospital, Hospices Civils de Lyon, Lyon, France; 2 Department of Systems Medicine, Gastroenterology and Endoscopy Unit, Tor Vergata University of Rome, Rome, Italy; 3 ClearSurgery SAS, Oullins, France

Digestive endoscopy practice is associated with various musculoskeletal disorders for the operator. This has been the subject of numerous studies and a guideline recently published by the American Society for Gastrointestinal Endoscopy 1 2 . Although these recommendations call for a neutral position for the operator, they are often difficult to implement in practice. Of particular concern are procedures performed under both fluoroscopic and endoscopic guidance, as they require the operator to alternate the attention between two monitors, often positioned on different axes relative to the right hand holding the endoscope. When the room is dedicated to endoscopic retrograde cholangiopancreatography (ERCP), where the operator’s neutral position is usually with their back to the patient, conventional procedures result in twisting movements of the knees, hips, back, and neck, with the latter two being at greatest risk of endoscopy-related injury 3 .

We report the case of a patient with esophageal stricture (eosinophilic esophagitis) referred for dilation using the BougieCap (Ovesco, Tübingen, Germany) technique under double fluoroscopic and endoscopic control. The procedure was performed in an ERCP room with a screen layout optimized for an operator with their back to the patient. The procedure therefore required a great deal of twisting on the part of the operator ( Fig. 1 **a** ). An augmented reality headset (ClearSurgery, Oullins, France) is a promising device for optimizing the ergonomics of gastrointestinal endoscopy, as it allows the operator to place the monitors in their field of view, whichever position is preferable, regardless of the environment in the room. In this example, the headset was held by an independent doctor not involved in the therapeutic procedure, as the system is currently undergoing the Conformité Européenne marking process ( Video 1 ). The use of this technique would allow the operator to perform the procedure in a neutral position, while allowing the nurses to place the screens at their convenience ( Fig. 1 **b** ).

**Fig. 1:**
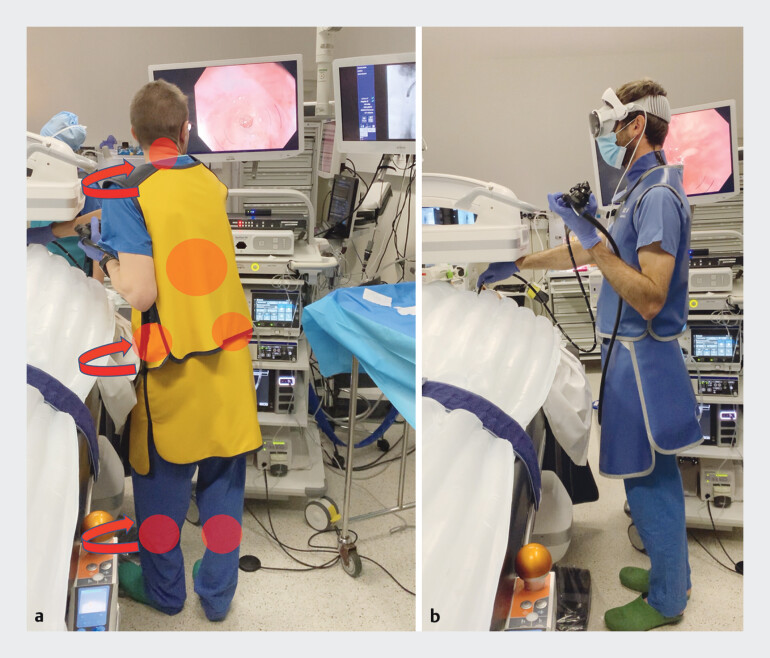
Operator positioning during endoscopy. **a** The conventional position of endoscopy screens in a room dedicated to endoscopic retrograde cholangiopancreatography forces the operator to make significant twisting movements when performing a procedure such as esophageal dilation. **b** The augmented reality headset allows the screens to be placed wherever the operator wishes in their field of view, to enable a neutral position.

Use of augmented reality digestive endoscopy to optimize ergonomics.Video 1

Although this augmented reality strategy is still undergoing approval, it seems a promising device for preventing endoscopy-related injuries by promoting optimal ergonomics for both the operator and the nurse, maintaining them in a neutral position and significantly improving the ergonomic aspect of the procedure.

Endoscopy_UCTN_Code_TTT_1AU_2AF

## Notes

Endoscopy E-Videos https://eref.thieme.de/e-videos
*E-Videos* is an open access online section of the journal *Endoscopy* , reporting on interesting cases and new techniques in gastroenterological endoscopy. All papers include a high-quality video and are published with a Creative Commons CC-BY license. Endoscopy E-Videos qualify for HINARI discounts and waivers and eligibility is automatically checked during the submission process. We grant 100% waivers to articles whose corresponding authors are based in Group A countries and 50% waivers to those who are based in Group B countries as classified by Research4Life (see: https://www.research4life.org/access/eligibility/ ). This section has its own submission website at https://mc.manuscriptcentral.com/e-videos .
